# Association of plasma amyloid-β oligomerization with theta/beta ratio in older adults

**DOI:** 10.3389/fnagi.2023.1291881

**Published:** 2023-12-01

**Authors:** Heewon Bae, Min Ju Kang, Sang-Won Ha, Da-Eun Jeong, Kiwon Lee, Seungui Lim, Jin-Young Min, Kyoung-Bok Min

**Affiliations:** ^1^Veterans Medical Research Institute, Veterans Health Service Medical Center, Seoul, Republic of Korea; ^2^Department of Clinical Research Design and Evaluation, Samsung Advanced Institute for Health Sciences and Technology, Sungkyunkwan University, Seoul, Republic of Korea; ^3^Department of Neurology, Veterans Health Service Medical Center, Seoul, Republic of Korea; ^4^Ybrain Research Institute, Seongnam-si, Republic of Korea; ^5^Department of Preventive Medicine, College of Medicine, Seoul National University, Seoul, Republic of Korea; ^6^Medical Research Center, Institute of Health Policy and Management, Seoul National University, Seoul, Republic of Korea

**Keywords:** amyloid oligomers, EEG, Alzheimer’s disease, cognitive impairment, neurotox

## Abstract

**Background:**

Oligomeric Aβ (OAβ) is a promising candidate marker for Alzheimer’s disease (AD) diagnosis. Electroencephalography (EEG) is a potential tool for early detection of AD. Still, whether EEG power ratios, particularly the theta/alpha ratio (TAR) and theta/beta ratio (TBR), reflect Aβ burden—a primary mechanism underlying cognitive impairment and AD. This study investigated the association of TAR and TBR with amyloid burden in older adults based on MDS-OAβ levels.

**Methods:**

529 individuals (aged ≥60 years) were recruited. All participants underwent EEG (MINDD SCAN, Ybrain Inc., South Korea) and AlzOn™ test (PeopleBio Inc., Gyeonggi-do, Republic of Korea) for quantifying MDS-OAβ values in the plasma. EEG variables were log-transformed to normalize the data distribution. Using the MDS-OAβ cutoff value (0.78 ng/mL), all participants were classified into two groups: high MDS-OAβ and low MDS-OAβ.

**Results:**

Participants with high MDS-OAβ levels had significantly higher TARs and TBRs than those with low MDS-OAβ levels. The log-transformed TBRs in the central lobe (β = 0.161, *p* = 0.0026), frontal lobe (β = 0.145, *p* = 0.0044), parietal lobe (β = 0.166, *p* = 0.0028), occipital lobe (β = 0.158, *p* = 0.0058), and temporal lobe (beta = 0.162, *p* = 0.0042) were significantly and positively associated with increases in MDS-OAβ levels. After adjusting for the Bonferroni correction, the TBRs in all lobe regions, except the occipital lobe, were significantly associated with increased MDS-OAβ levels.

**Conclusion:**

We found a significant association of MDS-OAβ with TBR in older adults. This finding indicates that an increase in amyloid burden may be associated with an increase in the low-frequency band and a decrease in the relatively high-frequency band.

## Introduction

Alzheimer’s disease (AD) is the most common cause of dementia, accounting for nearly 60–80% of all dementia cases. Clinically, AD is characterized by impairments in cognition and daily functioning (Deture and Dickson, 2019). Pathologically, the onset and progression of AD are characterized by the abnormal accumulation of amyloid-β (Aβ) plaques and neurofibrillary tangles in the brain (Beason-Held et al., 2013).

Oligomeric Aβ (OAβ) is a promising candidate marker for AD diagnosis (Youn et al., 2019). Serial cleavage of amyloid precursor proteins by β- and γ-secretase generates Aβ, first in its oligomeric and fibrous forms, ultimately leading to the generation of amyloid plaques (Sinha and Lieberburg, 1999). Among these, OAβ is the major neurotoxic species that leads to the development of AD (Tolar et al., 2021). Multimer Detection System-OAβ (MDS-OAβ), a blood test designed for detecting AD, measures the oligomerization tendency of amyloid-β in the blood. Accumulating evidence has demonstrated that MDS-OAβ levels are significantly associated with the presence and severity of cognitive impairment and that this association is stronger than that observed for Aβ plaques (Wang et al., 2017; Babapour Mofrad et al., 2021; Dominguez et al., 2022; Kim et al., 2022). Wang et al. (2017) reported that MDS-OAβ levels were well correlated with clinically used biomarkers of AD and exhibited good sensitivity (78.3%) and specificity (86.5%) in discrimination AD from healthy control cases. Another study noted that MDS-OAβ yielded good diagnostic accuracy (area under the curve [AUC]: 0.84–0.87) in distinguishing AD from control cases (Jeong, 2004; Wang et al., 2017; Colom-Cadena et al., 2020). In yet another study, MDS-OAβ levels could be used to identify individuals with abnormal amyloid PET findings, with an AUC of 0.77, which increased to 0.86 when combined with age and APOEe4 status. Collectively, these data suggest that plasma MDS-OAβ is a biomarker of brain amyloidosis (Jeong, 2004; Wang et al., 2017; Colom-Cadena et al., 2020; Babapour Mofrad et al., 2021; Dominguez et al., 2022; Kim et al., 2022).

Electroencephalography (EEG) is a noninvasive neuroimaging technique used to measure and record postsynaptic dendritic currents in synchronized cortical neurons (Frisoni et al., 2010). EEG has emerged as a potential tool for detecting aberrant neuronal activity associated with cognitive impairment and AD (Frisoni et al., 2010). Numerous lines of evidence have validated the possibility of using EEG for early detection of AD, reporting neural alterations in patients with AD such as decreased alpha and beta rhythm activity along with increased delta and theta oscillations (Babiloni et al., 2016; Cassani et al., 2018; Min et al., 2022). Ranasinghe et al. (2022) has shown that alpha and beta can be affected totally inverse way by amyloid accumulation and tau accumulation. Interestingly, in recent studies, EEG power ratios between fast and slow activities have been used to achieve good discrimination between different stages of AD (Bennys et al., 2001) Among these ratios, the theta/alpha ratio (TAR) and theta/beta ratio (TBR) are considered the most promising indices for AD diagnosis (Shulman and Goldstein, 2014; Tsolaki et al., 2014; Tzimourta et al., 2019). TAR can be sensitive and specific marker in early onset AD patients (Øzbek et al., 2021).Significant differences in the distribution of TAR and TBR have been observed between AD and healthy control groups, and multiple studies have documented an obvious visual separation between AD and healthy control groups with increasing window durations (Shulman and Goldstein, 2014; Tsolaki et al., 2014; Tzimourta et al., 2019). The results of these studies support the notion that TAR and TBR are potential markers for distinguishing patients with AD from controls (Brenner et al., 1986; Bennys et al., 2001).

Synaptic dysfunction and loss are fundamental to the pathological cognitive decline observed in AD (Colom-Cadena et al., 2020). Studies on human and mouse models have demonstrated that aggregated Aβ plaques are associated with local synapse loss, as well as losses in memory and synaptic plasticity (Koffie et al., 2012; Jackson et al., 2016), downstream of amyloidosis. EEG parameters represent changes in neuronal and synaptic dysfunction (Frisoni et al., 2010); however, it remains unknown whether EEG power ratios, particularly TAR and TBR, reflect Aβ burden—a major pathological mechanism underlying cognitive impairment and AD. Based on this circumstantial evidence, we hypothesized that elevation of TAR or TBR (i.e., the ratio of the increased relative power in the low-frequency band to the reduced relative power in the high-frequency band) is associated with a high amyloid burden. To examine this hypothesis, we investigated the association of TAR and TBR with amyloid burden in older adults, based on MDS-OAβ levels.

## Methods

### Study population

Between January and October 2022, 529 individuals (aged ≥60 years) were recruited from the Department of Neurology at the Veterans Health Service Medical Center in Seoul, Korea. All participants completed a neuropsychological test battery and underwent blood tests related to cognitive function, including the following: total, high-density lipoprotein (HDL), and low-density lipoprotein (LDL) cholesterol; triglycerides, free T4; thyroid stimulating hormone (TSH); and vitamin B12. A medical specialist interviewed all participants prior to each diagnosis.

The inclusion criteria were as follows: (a) self-report of cognitive decline; (b) ability to independently complete the clinical tests and questionnaires, and (c) voluntary agreement to participate in the study. The exclusion criteria were as follows: (a) diagnosis of brain infarction, cerebral hemorrhage, or Parkinson’s disease; and (b) diagnosis of another serious disease (e.g., cancer or mental illness). Experienced neurologic clinicians evaluated each patient based on the inclusion and exclusion criteria. The study protocols were approved by the Institutional Ethical Review Board of the Veterans Health Service Medical Center (BOHUN 2021–02-024, BOHUN 2022–05-006, BOHUN 2022–01-019).

The recruited participants underwent EEG and plasma MDS-OAβ measurements and completed baseline questionnaires (age, sex, education level, and assessments of depressive symptoms). A total of 529 individuals volunteered to participate in the study and provided informed consent upon enrollment. Of these, 102 participants subsequently dropped out of the study owing to incomplete responses related to diseases listed as exclusion criteria and refusal to undergo EEG or blood collection during examination. Thus, 427 individuals were eligible for inclusion.

### MDS-OAβ assay description and procedure

The AlzOn™ test (PeopleBio Inc., Gyeonggi-do, Republic of Korea) was employed to quantify MDS-OAβ values in the plasma samples from the subjects (An et al., 2017). This assay relies on the Multimer Detection System (MDS), a modified enzyme-linked immunosorbent assay (ELISA) that utilizes epitope-overlapping antibodies specific to the N-terminus of Aβ for the selective detection of Aβ oligomers over monomers in samples (An et al., 2010). Specifically, we used mouse monoclonal antibody 6E10 (BioLegend, San Diego, CA, United States) as the capturing antibody and WO2-HRP antibody (Absolute Antibody Ltd., Oxford, United Kingdom) as the detection antibody.

Blood samples were collected in BD vacutainer heparin tubes and centrifuged at 1500 X g for 10 min within 2 h of sampling. The obtained plasma was immediately aliquoted and stored at −80°C until analysis. Prior to the assay, aliquots of plasma samples were thawed at room temperature for 15 min. Following the assay protocol of the AlzOn™ test, HAMA blocker and PBR-1 (synthetic Aβ made by PeopleBio Inc.) was spiked into the plasma, and the mixture was incubated at 37°C for 48 h. The plasma sample mixture and serially diluted OAβ standards were then added to each well of 96-well plates precoated with capturing antibody and incubated at room temperature for 1 h. After washing the plates three times with washing buffer, HRP-conjugated detection antibody solution was added to the wells, and the plates were incubated for an additional hour at room temperature. Following another three washes with washing buffer, an enhanced chemiluminescence substrate solution was added, and the Relative Luminescence Unit (RLU) signal was measured using a microplate reader. Dilutions that provided signals within the linear range of the standard curves were used to convert RLU values into concentrations of oligomeric Aβ.

### Acquisition and analysis of EEG data

EEG assessments were conducted over 5 min in a quiet and soundproof room with dim lighting. The participants were in a comfortable resting state with their eyes closed. The EEG equipment used was a Ybrain MINDD-SCAN system (YEP-119B) with 19 channels and linked ears, which served as reference positions: Fp1, Fp2, F7, F3, Fz, F4, F8, T3, C3, Cz, C4, T4, T5, P3, Pz, P4, T6, O1, and O2. The impedance between the skin and all electrodes was consistently maintained at 5 kΩ or less. The sampling frequency was set to 500 Hz, and the resolution of the analog-to-digital converter was 24-bit. The collected data were stored and preserved in the European Data Format (EDF).

All preprocessing steps and analyses were conducted using Python (version 3.8.12). The EDF files were loaded using pyEDFlib (version 0.1.30), and the data recorded after the initial 5 s were processed using MNE (version 0.19.2). Basic filtering of the raw data was performed using an MNE notch filter (target: 60 Hz, notch width: 3 Hz) and a band-pass filter (low cutoff frequency: 1.3 Hz, high cutoff frequency: 60 Hz). Furthermore, a flat-line rejection and Random Sample Consensus (RANSAC) algorithm was applied to detect outliers and remove noisy channels.

The signal, which was now free of noise due to preprocessing, was converted to the frequency domain. The size of each band (delta: 1–4 Hz, theta: 4–8 Hz, alpha: 8–12 Hz, beta: 12–25 Hz, high beta: 25–30 Hz, gamma: 30–40 Hz) was averaged and multiplied by the size of the frequency range to calculate the absolute band power (uV2). To correct for individual deviations in absolute band power due to differences in impedance between the electrode and scalp during measurement, the relative band power (%) was calculated by dividing each band by the sum of all absolute bands 6. The band power ratios were calculated by dividing the values by the band power (DAR: delta/alpha ratio, TAR, TBR). The TAR and TBR were thus obtained by dividing the theta band power density by the alpha or beta band power density for each channel. The mean lobar TAR and TBR were defined as the sum of the corresponding channels in each lobe: FP1, FP2, F3, F4, F7, F8, and Fz leads for the frontal lobe; C3, C4, and Cz leads for the central lobe; T3, T4, T5, and T6 leads for the temporal lobe; P3, P4, and Pz leads for the parietal lobe; and O1 and O2 leads for the occipital lobe.

### Neuropsychological test

We used the brief version of the Seoul Neuropsychological Screening Battery (SNSB), named the SNSB-Core (SNSB-C), to evaluate cognitive performance (Kang et al., 2015). All participants completed tests including the Korean version of Boston Naming Test, Rey Complex Figure Test, Seoul Verbal Learning Test-Elderly’s version, Digit Symbol Coding, the Korean version of Color Word Stroop Test, Controlled Oral Word Association Test, and Korean-Trail Making Test-Elderly’s version.

### Statistical analysis

Using the MDS-OAβ cutoff value (0.78 ng/mL), all participants were classified into two groups: high MDS-OAβ (≥0.78 ng/mL) and low MDS-OAβ (<0.78 ng/mL) (Youn et al., 2020). The EEG indices (TAR and TBR) were log-transformed to normalize the data distribution. The Shapiro–Wilk normality test had a value of p greater than 0.05, which indicated normal distribution of data. Plasma levels of MDS-OAβ and the log- transformed EEG power ratio (TAR and TBR) in the five lobar regions (central, frontal, parietal, occipital, and temporal lobes) were compared between the two groups using t-tests. The correlation coefficients between plasma MDS-OAβ levels and the TAR and TBR in each of the five lobes were calculated using Pearson’s correlation analysis. We performed multiple linear regression to estimate the association between the log-transformed TAR or TBR in each lobe and plasma MDS-OAβ levels, with beta coefficients and standard error (SE) of the EEG power ratios. The regression models were adjusted for age, sex, and education level. In addition, we adjusted the Bonferroni correction to control the overall probability of a Type I error for multiple hypothesis tests.

All analyses were performed using Statistical Analysis System version 9.2 (SAS Institute, Cary, NC, United States), and the level of statistical significance was set at *p* ≤ 0.05.

## Results

Table 1 presents the characteristics of the overall study population and the two MDS-OAβ groups. Among the 427 participants, those age 75–79 accounted for the largest proportion (38.64%), and their average age was 74.9 years old. The proportion of male (55.74%) was higher than that of female. A high proportion of participants had below middle school education (43.56%), and the average number of educational years was 10.7 years. There were significant differences in age (*p* = 0.0109) and sex (*p* = 0.0454) between the low and high MDS-OAβ groups. In the low MDS-OAβ group, a high proportion of participants were 75–79 years old (n = 94; 42.34%), male (n = 134; 60.36%), and had below middle school education (n = 93; 41.89%). Their average age and educational year were 74.4 and 10.9 years, respectively. In the high MDS-OAβ group, a high proportion of participants were 75–79 years old (n = 71; 34.63%), male (n = 104; 50.73%), and had below middle school education (n = 93; 45.37%). Their average age and educational year were 75.4 and 10.5 years, respectively.

**Table 1:** Characteristics of the study population unit: *n* (%).

	Total population		MDS-OAβ (ng/mL)				
			Low (<0.78)		High (≥0.78)		*p*-value
60–64	21	(4.92)	11	(4.95)	10	(4.88)	0.0109
65–69	41	(9.60)	26	(11.71)	15	(7.32)	
70–74	127	(29.74)	67	(30.18)	60	(29.27)	
75–79	165	(38.64)	94	(42.34)	71	(34.63)	
80–84	58	(13.58)	18	(8.11)	40	(19.51)	
≥85	15	(3.51)	6	(2.70)	9	(4.39)	
Mean (SD) of Age	74.9	(5.30)	74.4	(5.02)	75.4	(5.55)	0.0413

Female	189	(44.26)	88	(39.64)	101	(49.27)	0.0454
Male	238	(55.74)	134	(60.36)	104	(50.73)	
Education level							
Below middle school	186	(43.56)	93	(41.89)	93	(45.37)	0.7696
High school	116	(27.17)	62	(27.93)	54	(26.34)	
Over college	125	(29.27)	67	(30.18)	58	(28.29)	
Mean (SD) of Education year 10.7	10.7	(4.46)	10.9	(4.41)	10.5	(4.51)	0.3658

Table 2 shows the mean (SD) of neuropsychological test scores according to MDS-OAβ cutoff value (0.78 ng/mL). The cognitive performance of older participants in the low MDS-OAβ group was significantly lower than that of the high MDS-OAβ group (all value of *p*<0.0001); 71.63 vs. 57.97 for Boston naming test, 48.44 vs. 24.80 for Rey copy test, 64.87 vs. 42.35 for Short-term verbal learning test, 73.18 vs. 50.24 for Digit symbol coding, 70.16 vs. 50.24 for Trail Making Test A, 73.65 vs. 56.64 for Trail Making Test B, and 62.91 vs. 35.83 for Color Word Stroop Test. Significant differences existed in Controlled Oral Word Association Test scores between the low and high MDS-OAβ groups. In particular, the score difference in the Controlled Oral Word Association Test (Animal+‘¬’) between the two groups (66.42 vs. 38.44) was considerable.

**Table 2:** Mean (SD) of neuropsychological test scores between the low and high MDS-OAβ groups.

Neuropsychological assessment	MDS-OAβ (ng/mL)				
	Low (<0.78)		High (≥0.78)		*p*-value
	Mean	SD	Mean	SD	
Boston naming test	71.63	(21.94)	57.97	(29.67)	<0.0001
Rey copy score	48.44	(19.39)	24.80	(23.58)	<0.0001
Short-term verbal learning test	64.87	(23.84)	42.35	(30.93)	<0.0001
Digit symbol coding	73.18	(22.99)	50.24	(28.89)	<0.0001
Controlled Oral Word Association Test					
- Animal	59.56	(24.37)	38.26	(29.34)	<0.0001
- ‘¬’	67.21	(25.83)	43.41	(28.08)	<0.0001
- Animal+‘¬’	66.42	(21.70)	38.44	(27.59)	<0.0001
Trail Making Test A	70.16	(17.83)	58.45	(24.06)	<0.0001
Trail Making Test B	73.65	(14.24)	56.64	(25.22)	<0.0001
Color Word Stroop Test	62.91	(25.12)	35.83	(27.66)	<0.0001

Table 3 shows the mean (SD) MDS-OAβ levels according to various characteristics of the study population. The mean MDS-OAβ level was elevated among older participants, female, and those with low education levels. In the low MDS-OAβ group, MDS-OAβ levels were higher in age 70–74 years, men, and older adults with below middle school education. In the high MDS-OAβ group, relatively higher MDS-OAβ levels were more likely for older participants, m, and those with low education levels.

**Table 3:** Mean (SD) MDS-OAβ by characteristics of the study population.

	Total population		MDS-OAβ (ng/mL)			
			Low (<0.78)		High (≥0.78)	
Age						
60–64	0.667	(0.30)	0.417	(0.21)	0.924	(0.09)
65–69	0.688	(0.31)	0.493	(0.20)	1.011	(0.13)
70–74	0.753	(0.27)	0.551	(0.19)	0.987	(0.14)
75–79	0.702	(0.29)	0.504	(0.21)	0.965	(0.14)
80–84	0.836	(0.30)	0.476	(0.22)	0.993	(0.15)
≥85	0.825	(0.25)	0.521	(0.21)	0.960	(0.12)
Sex						
Female	0.760	(0.28)	0.519	(0.19)	0.971	(0.28)
Male	0.718	(0.30)	0.505	(0.21)	0.980	(0.30)
Education level						
Below middle school	0.752	(0.28)	0.534	(0.19)	0.975	(0.14)
High school	0.719	(0.31)	0.475	(0.20)	0.982	(0.15)
Over college	0.731	(0.29)	0.511	(0.22)	0.970	(0.13)

MDS-OAβ: Multimer Detection System-Oligomeric Amyloid-β.

Table 4 shows the comparison of arithmetic mean (SD) EEG power ratios between the high and low MDS-OAβ groups according to lobar regions. In the total population, the TAR was highest in the frontal lobe (mean = 1.003, SD = 1.79), as was the TBR (mean = 0.821, SD = 1.80). Participants with high MDS-OAβ levels had higher mean TARs in all lobar regions than those with low MDS-OAβ levels, but there was no significant difference in TARs between them. In all lobar regions, the high MDS-OAβ group had a higher mean (SD) TBR than the low MDS-OAβ group: 0.843 (1.95) vs. 0.767 (1.74) in the central lobe (*p* = 0.0068), 1.041 (1.90) vs. 0.969 (1.68) in the frontal lobe (*p* = 0.0020), 0.840 (1.99) vs. 0.743 (1.82) in the parietal lobe (*p* = 0.0442), 0.902 (2.06) vs. 0.800 (1.82) in the occipital lobe (*p* = 0.0058), and 0.881 (2.06) vs. 0.778 (1.79) in the temporal lobe (*p* = 0.0044). Table 5 shows the correlation coefficients between the log-transformed EEG power ratio and MDS-OAβ by lobar regions. The correlation coefficients for the TBR were statistically significant in the central lobe (*r* = 0.133; *p* = 0.0061), frontal lobe (*r* = 0.122; *p* = 0.0117), parietal lobe (*r* = 0.132; *p* = 0.0064), occipital lobe (r = 0.122; *p* = 0.0114), and temporal lobe (*r* = 0.135; *p* = 0.0053); however, there were no significant correlations for the TAR. After adjusting for the Bonferroni correction, the log-transformed EEG power ratio was not significantly correlated.

**Table 4:** Arithmetic mean (SD) of EEG power ratios between the high and low MDS-OAβ groups, according to lobar regions.

EEG parameters	Total population		MDS-OAβ (ng/mL)				*p*-value
			Low (<0.78)		High (≥0.78)		
Theta-Alpha ratio							
Central lobe	0.802	(1.85)	0.645	(1.86)	0.742	(1.82)	0.5977
Frontal lobe	1.003	(1.79)	0.778	(1.80)	0.870	(1.78)	0.7549
Parietal lobe	0.788	(1.91)	0.554	(1.94)	0.644	(1.97)	0.7381
Occipital lobe	0.847	(1.94)	0.523	(2.00)	0.615	(2.04)	0.6826
Temporal lobe	0.826	(1.91)	0.680	(1.76)	0.768	(1.81)	0.4411
Theta-Beta ratio							
Central lobe	0.690	(1.85)	0.767	(1.74)	0.843	(1.95)	0.0068
Frontal lobe	0.821	(1.80)	0.969	(1.68)	1.041	(1.90)	0.0020
Parietal lobe	0.596	(1.96)	0.743	(1.82)	0.840	(1.99)	0.0442
Occipital lobe	0.566	(2.02)	0.800	(1.82)	0.902	(2.06)	0.0058
Temporal lobe	0.721	(1.79)	0.778	(1.79)	0.881	(2.03)	0.0044

MDS-OAβ: Multimer Detection System-Oligomeric Amyloid-β. EEG power ratios were log transformed.

**Table 5:** Correlation coefficients between the EEG power ratio and MDS-OAβ, according to lobar regions.

EEG parameters	*r*	*p*-value
Theta-Alpha ratio		
Central lobe	0.063	0.1972
Frontal lobe	0.047	0.3358
Parietal lobe	0.063	0.1969
Occipital lobe	0.060	0.2167
Temporal lobe	0.059	0.2235
Theta-Beta ratio		
Central lobe	0.133	0.0061
Frontal lobe	0.122	0.0117
Parietal lobe	0.132	0.0064
Occipital lobe	0.122	0.0114
Temporal lobe	0.135	0.0053

MDS-OAβ: Multimer Detection System-Oligomeric Amyloid-β. EEG power ratios were log transformed.

**Figure 1:**
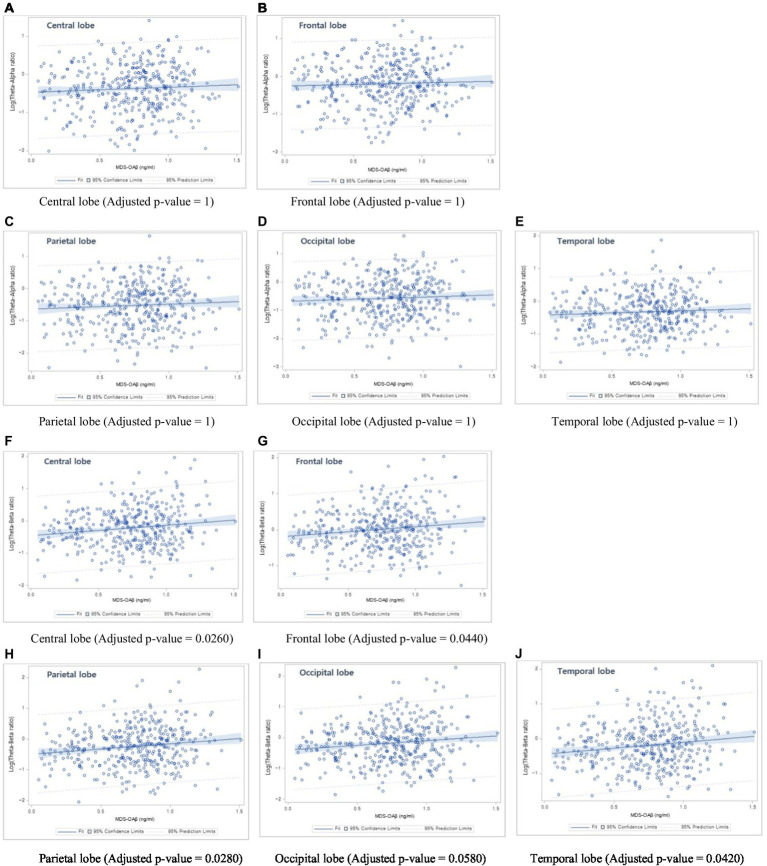
Linear regression plots. (1) Association between Theta-Alpha ratio and MDS-OAβ levels: **(A)** Central lobe, **(B)** Frontal lobe, **(C)** Parietal lobe, **(D)** Occipital lobe, and **(E)** Temporal lobe; (2) Association between Theta-Beta ratio and MDS-OAβ levels: **(F)** Central lobe, **(G)** Frontal lobe, **(H)** Parietal lobe, **(I)** Occipital lobe, and **(J)** Temporal lobe.

Table 6 shows the linear regression coefficients (SE) for log-transformed EEG power ratios associated with increased MDS-OAβ levels. The regression coefficient of the TAR was not significant for any lobar region. In contrast, the TBRs in the central lobe (β = 0.161, *p* = 0.0026), frontal lobe (β = 0.145, p = 0.0044), parietal lobe (β = 0.166, *p* = 0.0028), occipital lobe (β = 0.158, p = 0.0058), and temporal lobe (beta = 0.162, *p* = 0.0042) were significantly and positively associated with increases in MDS-OAβ levels. This association remained after adjusting for age, sex, and education level. After adjusting for the Bonferroni correction, the TBRs in all lobe regions, except the occipital lobe, were significantly associated with increased MDS-OAβ levels. Figure 1 displays the linear regression plots for the association of TAR and TAB in all lobar regions with MDS-OAβ levels.

**Table 6:** Linear regression coefficients for EEG power ratio associated with increased MDS-OAβ levels, according to lobar regions.

EEG parameters	Unadjusted model			Adjusted model^a^		
	β	(SE)	*p*-value	β	(SE)	*p*-value
Theta-Alpha ratio						
Central lobe	0.058	(0.06)	0.3007	0.044	(0.05)	0.4219
Frontal lobe	0.038	(0.05)	0.4682	0.031	(0.05)	0.5465
Parietal lobe	0.074	(0.06)	0.2258	0.046	(0.06)	0.4383
Occipital lobe	0.065	(0.06)	0.3076	0.044	(0.06)	0.4790
Temporal lobe	0.063	(0.05)	0.2277	0.046	(0.05)	0.3769
Theta-Beta ratio						
**Central lobe**	**0.170**	**(0.05)**	**0.0020**	**0.161**	**(0.05)**	**0.0026**
**Frontal lobe**	**0.154**	**(0.05)**	**0.0030**	**0.145**	**(0.05)**	**0.0044**
**Parietal lobe**	**0.181**	**(0.06)**	**0.0018**	**0.166**	**(0.06)**	**0.0028**
Occipital lobe	0.159	(0.06)	0.0073	0.158	(0.06)	0.0058
**Temporal lobe**	**0.175**	**(0.06)**	**0.0025**	**0.162**	**(0.06)**	**0.0042**

MDS-OAβ: Multimer Detection System-Oligomeric Amyloid-β. EEG power ratios were log transformed. Significant *p*-values after the Bonferroni correction are marked in bold.

a Adjusted by age, sex, and education level.

## Discussion

Our analysis revealed a significant association of MDS-OAβ with log-transformed EEG power ratios, TBR, among older adults. Specifically, individuals in the high MDS-OAβ group had significantly higher TBRs in the central, frontal, parietal, and temporal lobes than those in the low MDS-OAβ group. The observed association of MDS-OAβ with TBR indicates that an increase in amyloid burden may be associated with an increase in the low-frequency band and a decrease in the relatively high-frequency band.

AD is caused by the loss of synaptic junctions due to the accumulation of neurotoxic Aβ in the brain, and synaptic dysfunction is known to represent the best anatomical relationship for early cognitive impairment (Coben et al., 1985). In the amyloid cascade hypothesis, Aβ monomers are aggregated to form soluble Aβ oligomers (Babapour Mofrad et al., 2021). Some oligomers aggregate into amyloid plaques, while others cause neuronal damage, leading to dementia (Yagami, 2006). Among the various types of Aβ, OAβ is the most neurotoxic, and a strong correlation has been observed between its presence and the severity of cognitive symptoms, compared to the Aβ plaque burden (Meng et al., 2019; Babapour Mofrad et al., 2021). Aβ plaques deposited in the temporal neocortex in the early stages of AD interfere with neural circuits connecting the prefrontal cortex (Miao et al., 2021). Theta oscillations have been shown to orchestrate communication between prefrontal region and the hippocampus. Activation of the default mode network is inversely related to theta oscillations, and under-activation of the default mode network due to a high amyloid load ultimately results in hyperactivation of the midfrontal theta band (Schmidt et al., 2013). That is, an increase in theta and delta activity and a decrease in alpha and beta activity have been observed in the context of AD (Ingelsson et al., 2004; Sheng et al., 2012; Tsolaki et al., 2014; Walsh et al., 2017). Schmidt et al. (2013) identified a lower alpha/theta ratio in early and moderate stages of AD, indicating a pattern of increased theta and decreased alpha activity in patients with AD (Brenner et al., 1986). Previous studies have also demonstrated that patients with AD exhibit decreased beta power and increased theta power when compared with controls. Therefore, increased TAR and TBR values may be indicative of AD (Sheline et al., 2010).

Several studies have investigated the relationship between cerebrospinal fluid (CSF) biomarkers representing amyloid accumulation and changes in qEEG power spectral patterns. In one such study of 33 patients, Campbell et al. (2021) reported that the p-tau/Aβ42 and t-tau/Aβ42 ratios were strongly associated with increased relative theta power and reduced cognitive speed. A cross-sectional study of the INSIGHT-preAD cohort found increased theta band power in the middle prefrontal cortex in individuals with large amyloid deposits, relative to that observed in those with small amyloid deposits. This evidence suggests that theta power increase, one of the prominent markers of ‘EEG slowing’, is correlated with cortical amyloid deposition (Spinelli et al., 2022).

Our data are consistent with these findings and provide new insight, suggesting that increases in both TAR and TBR reflect Aβ pathology. These EEG changes may reflect amyloid-based hypoactivation of the default-mode network (Babiloni et al., 2018). Notably, we observed no significant association with Aβ oligomerization in the alpha band, a frequency strongly associated with AD progression. Our results suggest that TBR may be a better indicator than TAR. The MDS-OAβ method may reflect AD-induced changes in EEG; in particular, TBR may be more closely related to the amyloid burden.

The mechanism underlying the relationship between Aβ42 and slowing of EEG activity may be explained by cholinergic deficits. AD begins with alterations in signaling systems, including the cholinergic pathways. Studies in cultured rat cortical cells have demonstrated that amyloid peptides selectively impair the muscarinic cholinergic receptor-mediated signaling pathway (Spinelli et al., 2022). Aβ interferes with the mechanisms leading to uncoupling of muscarinic receptors and negatively affects acetylcholine synthesis and release. Decreased cortical cholinergic activity increases slow EEG activity (Huang et al., 2000). The negative effect of Aβ on cholinergic transmission in the brain can increase slow frequency activity (Spehlmann and Norcross, 1982; Visser et al., 2009). Alpha and beta waves are mainly generated in the cortex and propagate through connections within the cortex. Thus, brain atrophy in patients with AD may explain the rapid decline in the EEG rhythm (Smailovic et al., 2018). We hypothesize that decreased cholinergic activity, increased theta activity, and decreased beta activity due to brain atrophy are associated with the amyloid oligomerization pattern.

This study had several limitations. First, this study is based on cross-sectional, observational design. Second, while the biomarkers for brain amyloid have been well-established in cerebrospinal fluid amyloid-β 42 (Aβ42) (CSF Aβ42) and PET amyloid imaging, amyloid burden in our data was only based on plasma MDS-OAβ levels. MDS-OAβ is not a thoroughly validated marker for AD. However, owing to the high neurotoxicity of oligomerized Aβ, it has demonstrated a robust correlation with other clinical biomarkers, including CSF Aβ42 and amyloid PET. Third, the r-values of 0.133 indicate fairly weak correlation, but it’s still significant the value of conducting qEEG to detect toxic amyloid burden early in order to reduce the risk of AD.Longitudinal studies are necessary to confirm whether the TBR have prognostic potential for amyloid accumulation with amyloid PET imaging. Moreover, our population was individuals aged ≥60 years visited at the Department of neurology in a single medical center. Selection bias may be unavoidable, and our results cannot be generalized. Future work is needed to confirm the observed association from a representative sample and to reduce the uncertainty.

In conclusion, we found a significant association of MDS-OAβ with TBR among older adults. Increases in TBR were predominant among individuals with plasma MDS-OAβ levels. This contributes to research into EEG power ratio as a potential marker of AD, specifically linked to Aβ burden.

## Data availability statement

The raw data supporting the conclusions of this article will be made available by the authors, without undue reservation.

## Ethics statement

The studies involving humans were approved by Veterans Health Service Medical Center (IRB No. BOHUN 2021–02-024, BOHUN 2022–05-006, BOHUN 2022–01-019). The studies were conducted in accordance with the local legislation and institutional requirements. The participants provided their written informed consent to participate in this study.

## Author contributions

HB: Conceptualization, Methodology, Resources, Writing – original draft. MK: Methodology, Resources, Validation, riting – review & editing. S-WH: Investigation, Resources, Validation, Writing – review & editing. D-EJ: Data curation, Investigation, Resources, Writing – review & editing. KL: Data curation, Formal analysis, Resources, Software, Writing – review & editing. SL: Data curation, Formal analysis, Software, Writing – review & editing. J-YM: Conceptualization, Investigation, Methodology, Project administration, Resources, Supervision, Validation, Writing – original draft. K-BM: Conceptualization, Formal analysis, Funding acquisition, Investigation, Methodology, Project administration, Supervision, Writing – review & editing.

## References

[ref1] An S. S. A. Lee B. S. Yu J. S. Lim K. Kim G. J. Lee R.. (2017). Dynamic changes of oligomeric amyloid beta levels in plasma induced by spiked synthetic Abeta(42). Alzheimers Res. Ther. 9:86. doi: 10.1186/s13195-017-0310-6, PMID: 29041968 PMC5645921

[ref2] An S. S. Lim K. T. Oh H. J. Lee B. S. Zukic E. Ju Y. R.. (2010). Differentiating blood samples from scrapie infected and non-infected hamsters by detecting disease-associated prion proteins using Multimer detection system. Biochem. Biophys. Res. Commun. 392, 505–509. doi: 10.1016/j.bbrc.2010.01.053 20085753

[ref3] Babapour Mofrad R. Scheltens P. Kim S. Kang S. Youn Y. C. An S. S. A.. (2021). Plasma amyloid-beta oligomerization assay as a pre-screening test for amyloid status. Alzheimers Res. Ther. 13:133. doi: 10.1186/s13195-021-00873-w, PMID: 34311775 PMC8311929

[ref4] Babiloni C. Del Percio C. Lizio R. Noce G. Lopez S. Soricelli A.. (2018). Abnormalities of resting-state functional cortical connectivity in patients with dementia due to Alzheimer's and Lewy body diseases: an EEG study. Neurobiol. Aging 65, 18–40. doi: 10.1016/j.neurobiolaging.2017.12.023 29407464

[ref5] Babiloni C. Lizio R. Marzano N. Capotosto P. Soricelli A. Triggiani A. I.. (2016). Brain neural synchronization and functional coupling in Alzheimer's disease as revealed by resting state EEG rhythms. Int. J. Psychophysiol. 103, 88–102. doi: 10.1016/j.ijpsycho.2015.02.008, PMID: 25660305

[ref6] Beason-Held L. L. Goh J. O. An Y. Kraut M. A. O'brien R. J. Ferrucci L.. (2013). Changes in brain function occur years before the onset of cognitive impairment. J. Neurosci. 33, 18008–18014. doi: 10.1523/JNEUROSCI.1402-13.2013, PMID: 24227712 PMC3828456

[ref7] Bennys K. Rondouin G. Vergnes C. Touchon J. (2001). Diagnostic value of quantitative EEG in Alzheimer's disease. Neurophysiol. Clin. 31, 153–160. doi: 10.1016/S0987-7053(01)00254-4 11488226

[ref8] Brenner R. P. Ulrich R. F. Spiker D. G. Sclabassi R. J. Reynolds C. F. 3rd Marin R. S.. (1986). Computerized EEG spectral analysis in elderly normal, demented and depressed subjects. Electroencephalogr. Clin. Neurophysiol. 64, 483–492. doi: 10.1016/0013-4694(86)90184-7, PMID: 2430770

[ref1001] Campbell M. R. Ashrafzadeh-Kian S. Petersen R. C. Mielke M. M. Syrjanen J. A. Van Harten A. C.. (2021). P-tau/Aβ42 and Aβ42/40 ratios in CSF are equally predictive of amyloid PET status. Alzheimer’s Dement (Amst) 13:e12190. 34027020 10.1002/dad2.12190 PMC8129859

[ref9] Cassani R. Estarellas M. San-Martin R. Fraga F. J. Falk T. H. (2018). Systematic review on resting-state EEG for Alzheimer's disease diagnosis and progression assessment. Dis. Markers 2018, 1–26. doi: 10.1155/2018/5174815 PMC6200063 30405860

[ref10] Coben L. A. Danziger W. Storandt M. (1985). A longitudinal EEG study of mild senile dementia of Alzheimer type: changes at 1 year and at 2.5 years. Electroencephalogr. Clin. Neurophysiol. 61, 101–112. doi: 10.1016/0013-4694(85)91048-X, PMID: 2410219

[ref11] Colom-Cadena M. Spires-Jones T. Zetterberg H. Blennow K. Caggiano A. Dekosky S. T.. (2020). The clinical promise of biomarkers of synapse damage or loss in Alzheimer's disease. Alzheimers Res. Ther. 12:21. doi: 10.1186/s13195-020-00588-4, PMID: 32122400 PMC7053087

[ref12] Deture M. A. Dickson D. W. (2019). The neuropathological diagnosis of Alzheimer's disease. Mol. Neurodegener. 14:32. doi: 10.1186/s13024-019-0333-5, PMID: 31375134 PMC6679484

[ref13] Dominguez J. C. Yu J. R. T. De Guzman M. F. Ampil E. Guevarra A. C. Joson M. L.. (2022). Multimer detection system-Oligomerized amyloid Beta (MDS-OAbeta): a plasma-based biomarker differentiates Alzheimer's disease from other etiologies of dementia. Int. J. Alzheimers Dis. 2022:9960832. doi: 10.1155/2022/9960832 35547155 PMC9085320

[ref14] Frisoni G. B. Fox N. C. Jack C. R. Jr. Scheltens P. Thompson P. M. (2010). The clinical use of structural MRI in Alzheimer disease. Nat. Rev. Neurol. 6, 67–77. doi: 10.1038/nrneurol.2009.215, PMID: 20139996 PMC2938772

[ref15] Huang H. M. Ou H. C. Hsieh S. J. (2000). Amyloid beta peptide impaired carbachol but not glutamate-mediated phosphoinositide pathways in cultured rat cortical neurons. Neurochem. Res. 25, 303–312. doi: 10.1023/A:1007592007956 10786716

[ref16] Ingelsson M. Fukumoto H. Newell K. L. Growdon J. H. Hedley-Whyte E. T. Frosch M. P.. (2004). Early Abeta accumulation and progressive synaptic loss, gliosis, and tangle formation in AD brain. Neurology 62, 925–931. doi: 10.1212/01.WNL.0000115115.98960.37, PMID: 15037694

[ref17] Jackson R. J. Rudinskiy N. Herrmann A. G. Croft S. Kim J. M. Petrova V.. (2016). Human tau increases amyloid beta plaque size but not amyloid beta-mediated synapse loss in a novel mouse model of Alzheimer's disease. Eur. J. Neurosci. 44, 3056–3066. doi: 10.1111/ejn.13442, PMID: 27748574 PMC5215483

[ref18] Jeong J. (2004). EEG dynamics in patients with Alzheimer's disease. Clin. Neurophysiol. 115, 1490–1505. doi: 10.1016/j.clinph.2004.01.001 15203050

[ref19] Kang Y. Jahng S. Na D. (2015). Seoul neuropsychological screening battery-core (SNSB-C). Seoul: Human Brain Research & Consulting Co.

[ref20] Kim K. Y. Park J. Jeong Y. H. Kim H. J. Lee E. Park J. Y.. (2022). Plasma amyloid-beta oligomer is related to subjective cognitive decline and brain amyloid status. Alzheimers Res. Ther. 14:162. doi: 10.1186/s13195-022-01104-6, PMID: 36324157 PMC9632136

[ref21] Koffie R. M. Hashimoto T. Tai H. C. Kay K. R. Serrano-Pozo A. Joyner D.. (2012). Apolipoprotein E4 effects in Alzheimer's disease are mediated by synaptotoxic oligomeric amyloid-β. Brain 135, 2155–2168. doi: 10.1093/brain/aws127 22637583 PMC3381721

[ref22] Meng X. Li T. Wang X. Lv X. Sun Z. Zhang J.. (2019). Association between increased levels of amyloid-β oligomers in plasma and episodic memory loss in Alzheimer's disease. Alzheimers Res. Ther. 11:89. doi: 10.1186/s13195-019-0535-7, PMID: 31651358 PMC6814096

[ref23] Miao Y. Jurica P. Struzik Z. R. Hitomi T. Kinoshita A. Takahara Y.. (2021). Dynamic theta/beta ratio of clinical EEG in Alzheimer's disease. J. Neurosci. Methods 359:109219. doi: 10.1016/j.jneumeth.2021.109219, PMID: 34029602

[ref24] Min J. Y. Ha S. W. Lee K. Min K. B. (2022). Use of electroencephalogram, gait, and their combined signals for classifying cognitive impairment and normal cognition. Front. Aging Neurosci. 14:927295. doi: 10.3389/fnagi.2022.927295, PMID: 36158559 PMC9490417

[ref25] Øzbek Y. Fide E. Yener G. G. (2021). Resting-state EEG alpha/theta power ratio discriminates early-onset Alzheimer's disease from healthy controls. Clin. Neurophysiol. 132, 2019–2031. doi: 10.1016/j.clinph.2021.05.012, PMID: 34284236

[ref26] Ranasinghe K. G. Verma P. Cai C. Xie X. Kudo K. Gao X.. (2022). Altered excitatory and inhibitory neuronal subpopulation parameters are distinctly associated with tau and amyloid in Alzheimer’s disease. elife 11:e77850. doi: 10.7554/eLife.77850, PMID: 35616532 PMC9217132

[ref27] Schmidt M. T. Kanda P. A. Basile L. F. Da Silva Lopes H. F. Baratho R. Demario J. L.. (2013). Index of alpha/theta ratio of the electroencephalogram: a new marker for Alzheimer's disease. Front. Aging Neurosci. 5:60. doi: 10.3389/fnagi.2013.00060 24130529 PMC3793211

[ref28] Sheline Y. I. Raichle M. E. Snyder A. Z. Morris J. C. Head D. Wang S.. (2010). Amyloid plaques disrupt resting state default mode network connectivity in cognitively normal elderly. Biol. Psychiatry 67, 584–587. doi: 10.1016/j.biopsych.2009.08.024, PMID: 19833321 PMC2829379

[ref29] Sheng M. Sabatini B. L. Sudhof T. C. (2012). Synapses and Alzheimer's disease. Cold Spring Harb. Perspect. Biol. 4:a005777. doi: 10.1101/cshperspect.a005777, PMID: 22491782 PMC3331702

[ref30] Shulman A. Goldstein B. (2014). Electrophysiology quantitative electroencephalography/low resolution brain electromagnetic tomography functional brain imaging (QEEG LORETA): case report: subjective idiopathic tinnitus - predominantly central type severe disabling tinnitus. Int. Tinnitus J. 19, 10–27. doi: 10.5935/0946-5448.20140003 27186829

[ref31] Sinha S. Lieberburg I. (1999). Cellular mechanisms of beta-amyloid production and secretion. Proc. Natl. Acad. Sci. U. S. A. 96, 11049–11053. doi: 10.1073/pnas.96.20.11049, PMID: 10500121 PMC34239

[ref32] Smailovic U. Koenig T. Kareholt I. Andersson T. Kramberger M. G. Winblad B.. (2018). Quantitative EEG power and synchronization correlate with Alzheimer's disease CSF biomarkers. Neurobiol. Aging 63, 88–95. doi: 10.1016/j.neurobiolaging.2017.11.005, PMID: 29245058

[ref33] Spehlmann R. Norcross K. (1982). Cholinergic mechanisms in the production of focal cortical slow waves. Experientia 38, 109–111. doi: 10.1007/BF01944557, PMID: 7056349

[ref34] Spinelli G. Bakardjian H. Schwartz D. Potier M. C. Habert M. O. Levy M.. (2022). Theta band-Power shapes amyloid-driven longitudinal EEG changes in elderly subjective memory complainers at-risk for Alzheimer's disease. J. Alzheimers Dis. 90, 69–84. doi: 10.3233/JAD-220204, PMID: 36057818 PMC9661330

[ref35] Tolar M. Hey J. Power A. Abushakra S. (2021). Neurotoxic soluble amyloid oligomers drive Alzheimer's pathogenesis and represent a clinically validated target for slowing disease progression. Int. J. Mol. Sci. 22:6355. doi: 10.3390/ijms22126355, PMID: 34198582 PMC8231952

[ref36] Tsolaki A. Kazis D. Kompatsiaris I. Kosmidou V. Tsolaki M. (2014). Electroencephalogram and Alzheimer's disease: clinical and research approaches. Int. J. Alzheimers Dis. 2014:349249, 1–10. doi: 10.1155/2014/349249 24868482 PMC4020452

[ref37] Tzimourta K. D. Afrantou T. Ioannidis P. Karatzikou M. Tzallas A. T. Giannakeas N.. (2019). Analysis of electroencephalographic signals complexity regarding Alzheimer's disease. Comput. Electr. Eng. 76, 198–212. doi: 10.1016/j.compeleceng.2019.03.018

[ref38] Visser P. J. Verhey F. Knol D. L. Scheltens P. Wahlund L. O. Freund-Levi Y.. (2009). Prevalence and prognostic value of CSF markers of Alzheimer's disease pathology in patients with subjective cognitive impairment or mild cognitive impairment in the DESCRIPA study: a prospective cohort study. Lancet Neurol. 8, 619–627. doi: 10.1016/S1474-4422(09)70139-5 19523877

[ref39] Walsh C. Drinkenburg W. H. Ahnaou A. (2017). Neurophysiological assessment of neural network plasticity and connectivity: Progress towards early functional biomarkers for disease interception therapies in Alzheimer's disease. Neurosci. Biobehav. Rev. 73, 340–358. doi: 10.1016/j.neubiorev.2016.12.020, PMID: 28027953

[ref40] Wang M. J. Yi S. Han J. Y. Park S. Y. Jang J. W. Chun I. K.. (2017). Oligomeric forms of amyloid-beta protein in plasma as a potential blood-based biomarker for Alzheimer's disease. Alzheimers Res. Ther. 9:98. doi: 10.1186/s13195-017-0324-0, PMID: 29246249 PMC5732503

[ref41] Yagami T. (2006). Cerebral arachidonate cascade in dementia: Alzheimer's disease and vascular dementia. Curr. Neuropharmacol. 4, 87–100. doi: 10.2174/157015906775203011, PMID: 18615138 PMC2430680

[ref42] Youn Y. C. Kang S. Suh J. Park Y. H. Kang M. J. Pyun J. M.. (2019). Blood amyloid-beta oligomerization associated with neurodegeneration of Alzheimer's disease. Alzheimers Res. Ther. 11:40. doi: 10.1186/s13195-019-0499-7, PMID: 31077246 PMC6511146

[ref43] Youn Y. C. Lee B. S. Kim G. J. Ryu J. S. Lim K. Lee R.. (2020). Blood amyloid-β oligomerization as a biomarker of Alzheimer's disease: a blinded validation study. J. Alzheimers Dis. 75, 493–499. doi: 10.3233/JAD-200061, PMID: 32310175

